# A metformin add-on clinical study in multiple sclerosis to evaluate brain remyelination and neurodegeneration (MACSiMiSE-BRAIN): study protocol for a multi-center randomized placebo controlled clinical trial

**DOI:** 10.3389/fimmu.2024.1362629

**Published:** 2024-02-21

**Authors:** Anna-Victoria De Keersmaecker, Eline Van Doninck, Veronica Popescu, Lander Willem, Melissa Cambron, Guy Laureys, Miguel D’ Haeseleer, Maria Bjerke, Ella Roelant, Marc Lemmerling, Marie Beatrice D’hooghe, Judith Derdelinckx, Tatjana Reynders, Barbara Willekens

**Affiliations:** ^1^ Department of Neurology, Antwerp University Hospital, Edegem, Belgium; ^2^ Translational Neurosciences Research Group, Faculty of Medicine and Health Sciences, University of Antwerp, Edegem, Belgium; ^3^ Department of Family Medicine and Population Health, Faculty of Medicine and Health Sciences, University of Antwerp, Wilrijk, Belgium; ^4^ Center of Health Economic Research and Modelling Infectious Diseases, University of Antwerp, Wilrijk, Belgium; ^5^ Immunology and Infection, University of Hasselt, Diepenbeek, Belgium; ^6^ Biomedical Research Institute, University of Hasselt, Diepenbeek, Belgium; ^7^ Department of Neurology, Noorderhart Maria Hospital, Pelt, Belgium; ^8^ University Multiple Sclerosis Centre, University of Hasselt, Hasselt, Belgium; ^9^ Faculty of Medicine and Health Sciences, University of Ghent, Ghent, Belgium; ^10^ Department of Neurology, Algemeen Ziekenhuis Sint Jan, Bruges, Belgium; ^11^ Department of Neurology, University Hospital Ghent, Ghent, Belgium; ^12^ Department of Neurology, University Hospital Brussels, Brussels, Belgium; ^13^ Department of Neurology, National Multiple Sclerosis Center, Melsbroek, Belgium; ^14^ Department Neuroprotection and Neuromodulation, Center for Neurosciences, Faculty of Medicine and Pharmacy, Vrije Universiteit Brussel, Brussels, Belgium; ^15^ Neurochemistry Laboratory, Department of Clinical Biology, Brussels, University Hospital Brussels, Brussels, Belgium; ^16^ Department of Biomedical Sciences, University of Antwerp, Wilrijk, Belgium; ^17^ Clinical Trial Center, Antwerp University Hospital, Edegem, Belgium; ^18^ Department of Radiology, Antwerp University Hospital, Edegem, Wilrijk, Belgium; ^19^ Laboratory of Experimental Hematology, Vaccine and Infectious Disease Institute, Faculty of Medicine and Health Sciences, University of Antwerp, Wilrijk, Belgium

**Keywords:** progressive multiple sclerosis, metformin, remyelination, neurodegeneration, neuroprotection, clinical trial, repair

## Abstract

**Introduction:**

Despite advances in immunomodulatory treatments of multiple sclerosis (MS), patients with non-active progressive multiple sclerosis (PMS) continue to face a significant unmet need. Demyelination, smoldering inflammation and neurodegeneration are important drivers of disability progression that are insufficiently targeted by current treatment approaches. Promising preclinical data support repurposing of metformin for treatment of PMS. The objective of this clinical trial is to evaluate whether metformin, as add-on treatment, is superior to placebo in delaying disease progression in patients with non-active PMS.

**Methods and analysis:**

MACSiMiSE-BRAIN is a multi-center two-arm, 1:1 randomized, triple-blind, placebo-controlled clinical trial, conducted at five sites in Belgium. Enrollment of 120 patients with non-active PMS is planned. Each participant will undergo a screening visit with assessment of baseline magnetic resonance imaging (MRI), clinical tests, questionnaires, and a safety laboratory assessment. Following randomization, participants will be assigned to either the treatment (metformin) or placebo group. Subsequently, they will undergo a 96-week follow-up period. The primary outcome is change in walking speed, as measured by the Timed 25-Foot Walk Test, from baseline to 96 weeks. Secondary outcome measures include change in neurological disability (Expanded Disability Status Score), information processing speed (Symbol Digit Modalities Test) and hand function (9-Hole Peg test). Annual brain MRI will be performed to assess evolution in brain volumetry and diffusion metrics. As patients may not progress in all domains, a composite outcome, the Overall Disability Response Score will be additionally evaluated as an exploratory outcome. Other exploratory outcomes will consist of paramagnetic rim lesions, the 2-minute walking test and health economic analyses as well as both patient- and caregiver-reported outcomes like the EQ-5D-5L, the Multiple Sclerosis Impact Scale and the Caregiver Strain Index.

**Ethics and dissemination:**

Clinical trial authorization from regulatory agencies [Ethical Committee and Federal Agency for Medicines and Health Products (FAMHP)] was obtained after submission to the centralized European Clinical Trial Information System. The results of this clinical trial will be disseminated at scientific conferences, in peer-reviewed publications, to patient associations and the general public.

**Trial registration:**

ClinicalTrials.gov Identifier: NCT05893225, EUCT number: 2023-503190-38-00.

## 1 Introduction

World-wide around 2.8 million people are affected by multiple sclerosis (MS), a common and disabling immune-mediated and neurodegenerative disease of the central nervous system (CNS) which results in focal and diffuse demyelination and neuroaxonal damage (1). Onset of disease happens during (young) adulthood in the majority of cases (2, 3) and the subsequent disease course may be relapsing-remitting (RRMS) or progressive (PMS). There is no strict cut-off point between relapsing and progressive MS, leading to a recently suggested redefining of the disease course in a more biological manner (4–6). Neurodegeneration is believed to be triggered by sustained demyelination and an exhaustion of compensatory mechanisms, leading to a cascade of events including oxidative stress, mitochondrial injury, energy failure and ionic dysregulation (7). Despite the numerous immunomodulating therapies for MS that have proven benefits in reducing inflammatory disease processes, a considerable number of individuals experience disability progression. This is also referred to as silent progression, smoldering MS, or more recently, progression independent of relapse activity (PIRA). More than relapse-associated worsening, PIRA is considered a major contributor to long-term disability accumulation in MS. PIRA has been associated with brain and spinal cord atrophy and chronic active lesions on MRI (8–12). Paramagnetic rim lesions (PRLs) and slowly expanding lesions (SELs) are markers of chronic active lesions, associated with accumulation of tissue damage. PRLs are visualized using susceptibility weighted images (SWI) based on the presence of iron-containing macrophages and/or microglia at the lesion edge. SELs are detected on longitudinal T1 and T2 weighted images and correspond to non-enhancing T2 lesions with greater microstructural damage and ongoing inflammation. Co-localization of these lesions corresponds with the most destructive type of chronic lesions (13, 14). PMS places a significant hardship on patients, not only by diminishing their quality of life and limiting their involvement in the workforce and community activities, but also by imposing a burden on the healthcare system (cost) and caregivers (strain) (10, 15, 16).

Currently approved disease-modifying therapies (DMTs) mainly focus on controlling the malfunctioning adaptive immune responses that are driving the early disease process but are unable to halt disease progression in PMS. High-efficacy DMTs such as anti-CD20 treatments do not completely resolve PRLs, which are regarded as surrogate markers for chronic active lesions in multiple sclerosis (17). In fact, multiple highly relevant PMS disease related mechanisms, such as chronic microglial inflammation, oxidative stress and failure of remyelination remain unaddressed by currently approved DMTs (11, 18). Two approved DMTs for active PMS, ocrelizumab and siponimod, do exhibit enhanced effectiveness in patients with ongoing disease activity. However, their impact on clinical disease progression remains limited, yielding reductions of only 24% and 21% respectively when compared to a placebo (19). Promising results have been observed in a clinical study with alpha lipoic acid and high dose simvastatin, while other clinical trials investigating treatment with Bruton’s tyrosine kinase inhibitors (BTKis) in PMS are ongoing (20). However, recently it was announced that two phase III clinical trials of the BTKi evobrutinib versus teriflunomide in RMS did not meet their primary endpoints, dampening the high hopes for the clinical use of evobrutinib and BTKis in general to treat MS. Results of clinical trials investigating other BTKis are therefore eagerly awaited. Several other therapeutic approaches explored in the past, such as amiloride, fluoxetine, rilozole, and high-dose biotin, were proven unsuccessful (21–24). The unmet and urgent medical need for a neuroprotective and restorative treatment in PMS patients is clear and well recognized by the international research community as specified in a perspective by the Progressive MS Alliance (25).

To tackle this major unmet need, remyelination, which leads to restoration of axonal conduction and holds the key for clinical recovery in MS, is an interesting focus. Remyelination is an endogenous process that occurs naturally, but that declines with ageing and is compromised in the context of MS. In a healthy CNS, oligodendrocyte precursor cells (OPCs) generate myelin-producing oligodendrocytes (ODCs), which are responsible for forming the myelin sheaths around axons. However, within MS lesions, OPCs are present but fail to differentiate and form new myelin sheaths, resulting in the unveiling of the underlying axonal membrane, subsequently causing a disruption in axonal conduction. In demyelinated axons, sodium channels are redistributed resulting in an influx of extracellular sodium and forcing the energy-demanding Na/K pump to work harder to maintain proper ionic balance and cell polarization. To generate this energy, axons highly depend on mitochondria. However, inflammatory processes involving immune cells producing reactive oxygen species (ROS) and nitric oxide (NO), severely affect the integrity of mitochondria. In the end, the imbalance between heightened energy demands and reduced mitochondrial metabolic function can initiate certain processes that ultimately lead to neurodegeneration (7, 26).

Hence, stimulation of endogenous remyelination may have a significant therapeutic impact, since it will safeguard the axons from being damaged (27–29). In this context, metformin, traditionally used in the treatment of patients with type 2 diabetes mellitus, is a promising agent. It has been put forward as a potential anti-ageing drug, due to its pleiotropic properties, among which antioxidant, antiproliferative, antifibrotic and immunomodulatory effects have been demonstrated (30–32).

In the last two decades, metformin has been investigated in rodent models of MS and demyelination, including the MOG-induced Experimental Autoimmune Encephalomyelitis (EAE)- and cuprizone- model (33–35). In the EAE model, metformin has proven its ability to reduce inflammation, improve functional outcomes of disease symptoms and decrease demyelination (36). Additionally, functioning as an agonist of AMP-activated protein kinase (AMPK), the drug has exhibited protective effects on OPCs and ODC lineage dynamics (37, 38). One of the most compelling preclinical studies to support the use of metformin in MS investigated the effects of ageing on OPCs. When aged OPCs were transplanted into neonatal CNS of rats, they exhibited the same proliferation capabilities as neonatal OPCs, while conversely, neonatal OPCs ceased to proliferate when transplanted into aged rat brains. This suggests that, rather than intrinsic OPCs-factors, it is the local environment that impacts OPCs function. Therefore, rejuvenating the environment of OPCs may lead to improved remyelination. One well known modulator of ageing is fasting and when aged animals are subjected to a fasting regime, remyelination capacities comparable with those seen in young animals can be observed. Although intermittent fasting was deemed to be difficult in clinical practice, researchers found that the effect in the described experiment could be perfectly mimicked through the use of metformin. Indeed, metformin rejuvenated the OPCs and made them responsive to differentiation factors once again, allowing them to proliferate (10, 39–41). The importance of AMPK signaling, leading to improved mitochondrial homeostasis and protection of ODCs, was confirmed in the cuprizone model. A recent study discovered that metformin led to a dose dependent increase in AMPK activation in healthy ODCs, which resulted in both promotion of changes in ODC bioenergetics and acceleration of ODC differentiation. A knockdown approach in this study confirmed the findings of Neumann et al. (39) that metformin does need an active AMPK complex in order to promote OPC differentiation. Additional *in vivo* work in this study confirmed the enhanced ODC differentiation and remyelination promotion in a cuprizone model, particularly in white matter regions (38). Additional mechanisms of metformin include antioxidant mechanisms related to the mammalian target of rapamycin (mTOR) pathway and the nuclear factor erythroid 2-related factor 2 (Nrf2) pathway, as well as reduced microgliosis by down-regulation of Mac-3 mRNA, a marker of pro-inflammatory microglia/macrophages (42–44).

Based on preclinical evidence, metformin has shown neuroprotective effects in other neurodegenerative disorders (Parkinson’s disease, Alzheimer’s disease, Huntington disease). Metformin is able to cross the blood brain barrier and exert anti-apoptotic, antioxidant and regulatory effects on neuroinflammation, microglial activity, brain derived neurotrophic factor mediation, neurotransmission and neurogenesis (45, 46). A clinical study within a cohort of individuals diagnosed with type 2 diabetes mellitus explored the impact of metformin treatment on the likelihood of developing dementia. In this population, treatment with metformin could be linked to a dose-dependent decrease in the risk of dementia (47). Another population-based cohort study confirmed these findings: individuals with type 2 diabetes initiating metformin exhibited a markedly reduced risk of dementia compared to individuals who were not prescribed any anti-diabetes medication, even though the latter group had superior glycemic profiles and cardiovascular health at the study’s onset (48).

In addition, promising results of metformin use in patients with MS have been revealed through clinical research. In a pilot study in adult obese MS patients with metabolic syndrome (N = 50), treatment with metformin (dose range of 850-1500 mg/day) led to a reduction in new T2 hyperintense brain lesions on MRI in 20 patients. This positive impact was accompanied by immunological changes, supporting an anti-inflammatory effect of metformin in MS. Notably, the treatment was well tolerated (49). In a cross-sectional study investigating comorbidities in MS (N = 453), it was noted that diabetes type 2/insulin resistance was associated with a reduced risk of disability (Expanded Disability Status Score (EDSS) ≥ 6.0) in 87 PMS patients. Interestingly, 72% of these individuals were using metformin as therapy (50). It is important to highlight that vascular comorbidities, such as diabetes, are associated with clinical and imaging markers of both neurological dysfunction and neurodegeneration in MS patients (51). However, despite the unfavorable prognoses associated with these comorbidities, metformin demonstrated positive effects in this specific subset of MS patients. Another exploratory prospective single-center, phase II, randomized, open-label clinical trial investigated the safety and efficacy of metformin as adjuvant to interferon-beta-1a treatment in relapsing remitting MS patients during one year. The study revealed a statistically significant change in serum malondialdehyde, an indicator of oxidative stress. However, no significant effects were found for additional immunological, imaging (T2 lesions) and clinical (EDSS) outcome measures (52). It is well known that EDSS is less sensitive to change than the Timed 25 Foot Walk test (T25FW) in order to detect disability progression in PMS (53). The sensitivity of T25FW as a clinical outcome measure for capturing intervention effects on progression in MS in pharmacological and rehabilitation trials has been confirmed in various clinical trials (54–57).

Several other clinical trials are currently ongoing and are summarized in **Table 1**. One single-center, phase I, randomized clinical trial in Canada is recruiting pediatric and young adult MS patients to investigate safety, feasibility and visual outcome measures of metformin add-on treatment. Another single center, phase IIa, randomized placebo-controlled clinical trial will evaluate the effect of combination therapy of clemastine 1,34 mg prolonged release tablets with metformin 500 mg prolonged release tablets on safety, visual evoked potentials and MRI outcome measures, in patients with relapsing MS who are on a disease-modifying therapy. In the USA a single-center, phase I, placebo-controlled, randomized clinical trial in PMS patients has started recruiting patients. Here they investigate the safety and efficacy of metformin add-on treatment. A large phase II/III multi-center, multi-stage, multi-arm, randomized, placebo-controlled clinical trial (OCTOPUS) will investigate efficacy and safety of multiple drugs (including metformin and alpha-lipoic acid) in both primary and secondary progressive patients. The main outcomes are whole brain atrophy on MRI and disability progression. In Australia, the PLATYPUS study will follow the same protocol as the OCTOPUS clinical trial.

**Table 1:** Summary of clinical trials investigating the safety and/or efficacy of metformin as treatment for MS.

Clinical trial identifier	Title	Sample size	Info, arms and intervention	Outcome measures	Status
NCT04121468	A Phase I Double Blind Study of Metformin Acting on Endogenous Neural Progenitor Cells in Children With Multiple Sclerosis (Canada)	30 (age 10-25)	Single center. Duration 12 months. 3 groups will start taking metformin at different timepoints (3, 6 and 9 months). In remaining time participants will take a placebo. Dose may be increased up to a maximum of 2000 mg/day. Patients need to have involvement of the anterior visual pathways and stable immunomodulatory treatment.	Primary: safety and feasibility Secondary: visual evoked potentials and optical coherence tomography	Recruiting
NCT05131828	CCMR Two: A Phase IIa, Randomized, Double-blind, Placebo-controlled Trial of the Ability of the Combination of Metformin and Clemastine to Promote Remyelination in People With Relapsing-remitting Multiple Sclerosis Already on Disease-modifying Therapy (UK)	50 (age 25-50)	Single center. Duration 24 weeks. Evaluation of metformin 500 mg prolonged released tablets combined with clemastine 1,34 mg prolonged release tablets, in patients with RRMS who are on a disease-modifying therapy.	Primary: based on visual evoked potentials Secondary: related to safety and MRI	Recruiting
NCT05349474	Metformin Treatment in Progressive Multiple Sclerosis (USA)	44 (age 30-65)	Single center. Duration 12 months. Assessing the safety of metformin 500mg oral tablets up to 4 tablets per day, in comparison to placebo in patients with PMS who are on stable disease-modifying therapy.	Primary: safety assessments (including number of adverse events, laboratory abnormalities and new T2 lesions) Secondary: potential efficacy (MRI and clinical outcome measures)	Recruiting
NCT05298670	Drug Repurposing Using Metformin for Improving the Therapeutic Outcome in Multiple Sclerosis Patients (Egypt)	80 (age 18-50)	Single center. Duration 6 months. Evaluation of treatment with interferon-beta 1a (comparator) or interferon-beta 1a + metformin 1000mg in patients with RRMS.	Primary: change in interleukin-17 levels Secondary: limited clinical and MRI assessments	Completed (52)
ISRCTN 14048364	OCTOPUS: Optimal Clinical Trials Platform for Multiple Sclerosis (UK)	1200 (age 25-70)	Multi center. Duration up to 5 years. Phase II/III clinical trial that will use MAMS design (multi-arm, multi-stage), randomized, placebo-controlled. Will assess the efficacy and safety/tolerability of multiple drugs (including metformin and alpha-lipoic acid).	Primary, stage I: MRI (brain atrophy) Primary, stage II: time to disability progression via composite endpoint	Recruiting
NCT05893225	MACSiMiSE-BRAIN: Metformin Add-on Clinical Study in Multiple Sclerosis to Evaluate Brain Remyelination And Neurodegeneration (Belgium)	120 (age 18-70)	Multi center. Duration 96 weeks Evaluation of add-on treatment with Metformin in comparison with placebo in patients with PMS.	Primary: change in T25FWT Secondary: EDSS, 9HPT, SDMT, MRI and DTI Exploratory: Patients reported outcomes, Iron rim lesions, cost-effectiveness	Recruiting
T.B.A.	PLATYPUS (Australia)	250 (age 25-70)	Multi center. Extension of OCTOPUS (UK). Duration up to 5 years. Phase II/III clinical trial that will use MAMS design (multi-arm, multi-stage), randomized, placebo-controlled. Will assess the efficacy and safety/tolerability of multiple drugs (including metformin and alpha-lipoic acid).	Primary, stage I: MRI (brain atrophy) Primary, stage II: time to disability progression via composite endpoint	Not yet recruiting

In conclusion, the time is right to start a clinical trial which can confirm preliminary but promising findings from human studies by evaluating the efficacy of metformin in PMS.

## 2 Methods and analysis

### 2.1 Study design

A multicenter two arm, 1:1 randomized, triple-blind, placebo-controlled clinical trial comparing metformin versus placebo in patients with non-active PMS will be conducted at 5 sites in Belgium. Participants with PMS, along with caregivers and investigators, will be blinded. We hypothesize that metformin, as add-on treatment in patients with PMS, is able to delay disease progression in comparison to placebo.

Patient recruitment has started in December 2023. After meeting the inclusion criteria and providing written informed consent, a total of 120 patients will be included and randomized 1:1 to either the metformin or placebo group. The randomization will be stratified. The sample size calculation was based on the primary outcome measure, the change in walking speed as measured by the Timed 25-Foot Walk Test (T25FW). The sample size was calculated based on previously reported mean T25FW measurements in PMS patients of 11.2 and 11.5 seconds (22, 58) and the assumption that a 20% change in T25FW is seen as clinically relevant, which amounts to a decrease of on average 2 seconds over 96 weeks (59–62). Using the annualized rate of change described by Spain et al. (58), we estimated a standard deviation (SD) on the change in T25FW from baseline to 96 weeks of treatment of 3.4 s. Assuming a SD of 3.4 s and a significance level of 0.05, an achieved sample size of 47 per group is required to detect a reduction of 2 s in the change from baseline to 96 weeks comparing metformin to placebo with 80% power using a two-sample t-test (63). A sample size of 59 per arm will allow for a 20% drop-out rate.

**Figure 1:**
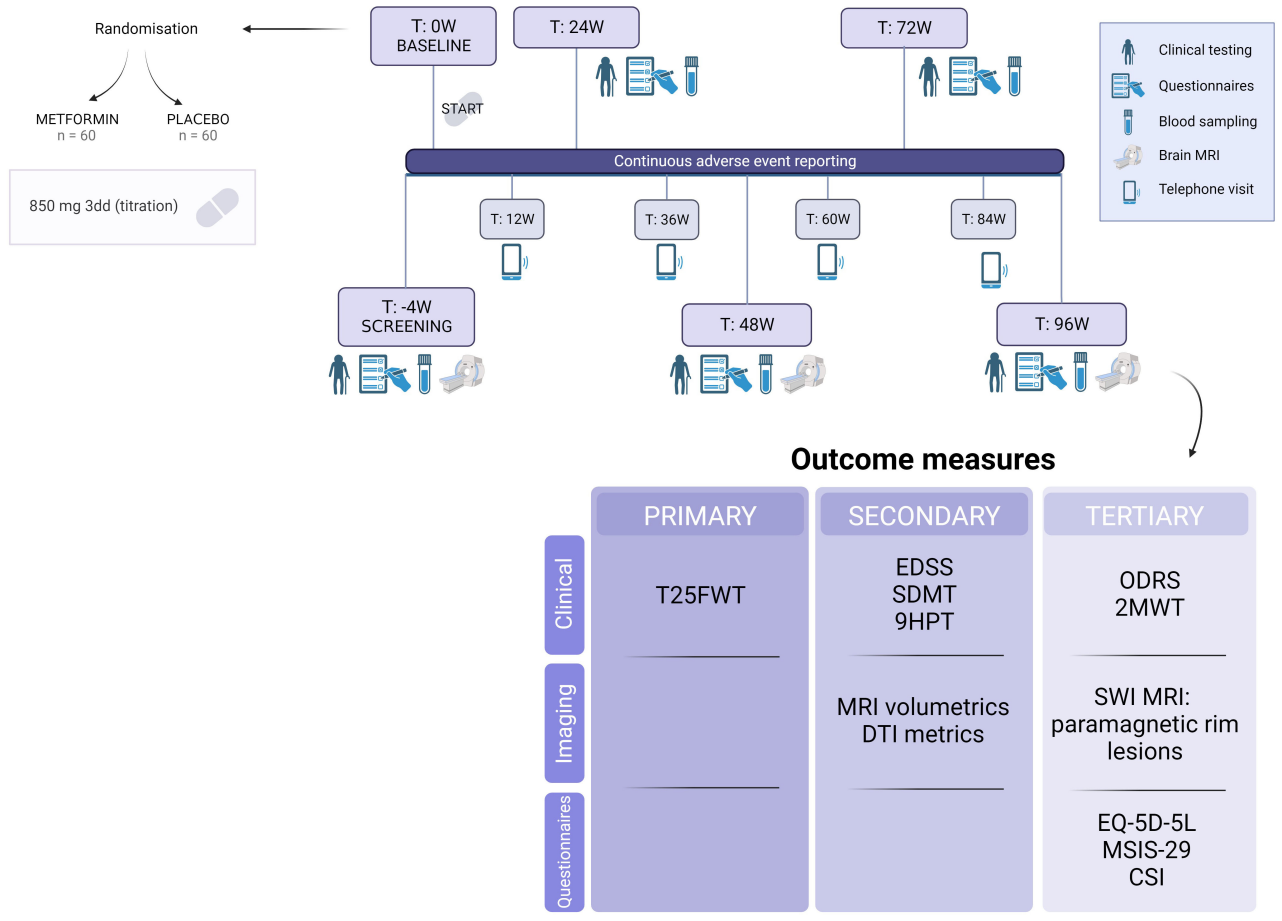
Timeline and outcome measures of the MACSiMiSE-BRAIN clinical trial. See **Additional File**. Created with BioRender.com.

Patients will complete 6 onsite study visits and 4 remote telephone visits over the course of a 100-week time period (96 weeks of treatment plus 4 weeks for screening), starting with a screening visit (**Figure 1**). Caregivers will also be given the opportunity to engage in the study, by providing information on caregiver strain at the same timepoints as the patient’s onsite visits. Inclusion of caregivers is only possible after a separate signed informed consent. The anticipated conclusion of the study is set for the end of 2026. A more detailed study schedule which also includes substudies can be found in **Supplementary Table 3**

### 2.2 Objectives

The primary objective is to determine whether treatment with metformin is able to delay disease progression in patients with non-active PMS when compared to placebo, by the means of T25FW measurements. A secondary objective is to assess the delay in disease progression across several other affected domains, including MS-related disability, hand function, and cognitive function. Moreover, we intend to investigate the biological efficacy through brain MRI volumetry and brain MRI Diffusion Tensor Imaging (DTI) metrics (64–67). As an exploratory objective we hypothesize that treatment with metformin will exhibit a correlation with changes in paramagnetic rim lesions. Additional tertiary objectives encompass: (i) demonstrating improved quality of life for patients, (ii) showing decrease in caregiver strain and (iii) proving cost-effectiveness of this approach (health-economic analysis).

### 2.3 Study population

#### 2.3.1 Eligibility and enrolment

Patients with non-active PMS, diagnosed according to the 2017 revised McDonald criteria (5) and the disease course definitions of Lublin (68), evidenced by PIRA and the absence of relapses and/or new T2-lesions and/or enhancing T1-lesions in the past year (NEDA-2), will be recruited (5, 68). Patients are included after giving written informed consent and finally enrolled in the clinical trial when the inclusion and exclusion criteria are met (**Table 2**).

**Table 2:** List of inclusion- and exclusion-criteria for enrollment.

Inclusion criteria	Exclusion criteria
▪ A diagnosis of non-active progressive multiple sclerosis (including PPMS and SPMS, in accordance with 2017 revisions of McDonald criteria (5) and disease course definitions of Lublin 2013 (68)), as evidenced by: a. the absence of relapses and new T2 lesions and/or enhancing T1 lesions on brain MRI in the past 1- 2 years or longer (NEDA-2) b. progression of disability independent of relapses in the past 1-2 years or longer. If progression is defined as one of the following, over the past 1-2 years or less, the patient can be included without additional review: o minimum increase in the EDSS of 1.0, or 0.5 from a baseline level of 2.0–5.0, and 5.5- 6.0, respectively o ≥20% in the T25W o ≥20% 9HPT o reduction of ≥4 points or a 10% worsening in the Symbol Digit Modality Test without concomitant depression or fatigue ▪ If the investigator is in the opinion that the patient is clearly progressing, but not enough data are available to demonstrate this, a narrative need to be provided, which will be judged by at least 2 members of the trial steering committee, from a center that is not submitting the case for review. ▪ Age 18-70 years inclusive ▪ EDSS 2.0-6.5 inclusive ▪ Able to give informed consent (signed, written) and to adhere to study procedures ▪ Dutch/Flemish speaking ▪ Stable use of DMT or no treatment in the past year or longer ▪ Use of adequate contraceptive measures in women of childbearing potential (WOCBP); no contraception is required for male subjects with pregnant or non-pregnant WOCBP partner.	▪ Diagnosis of diabetes mellitus or fasting glucose level of 126mg/dl or more; random glucose level of 200mg/dl or more; HbA1C of 6.5% or more at screening ▪ A medical or neurological problem other than MS that is a cause of progressive or fluctuating gait dysfunction ▪ Unable to complete T25FW ▪ Unable to undergo MRI ▪ Current major disease or disorder other than MS (e.g., active malignancy, significant renal insufficiency eGFR 3 times ULN, chronic active infection etc.) that may interfere with study procedures and/or intake of study drug ▪ Pregnant or breast-feeding or planning pregnancy ▪ Use of an experimental therapy in the past 6 months ▪ Ongoing immune reconstitution therapy schedule (cladribine second course ended at least 12 months before inclusion, alemtuzumab second/last course at least 12 months before inclusion, AHSCT at least 12 months before inclusion) ▪ Expected change in ongoing DMT or start of DMT if untreated ▪ Current use of metformin or known intolerance for metformin ▪ Known sensitivity to active substance or to any or the excipients ▪ All forms of acute metabolic acidosis (such as lactic acidosis, diabetic ketoacidosis, diabetic precoma ▪ Acute conditions where there is a risk of alteration of renal function, such as: dehydration, severe infection, shock occurring between screening and randomization ▪ Chronic use of NSAID

During the clinical trial it is requested not to start fampridine or 4-aminopyridine (or keep the dose stable), keep physical therapy use stable and not change the DMT for MS, when the medical condition of the patient allows it.

### 2.4 Study medication and dose

Metformin hydrochloride is a generic, FDA and EMA approved drug for treatment of patients with diabetes mellitus type 2. Its adverse events, interactions, risks and precautions are well-known. Metformin Hydrochloride 850 mg will be purchased as product with marketing authorization (MA) and repackaged. Matching placebo tablets will be produced under GMP conditions and distributed by a licensed company (Ardena). Taking into account doses used in preclinical studies, the maximum daily dose of 3000 mg, considerations of tolerance and available tablet dosing, the suggested daily dose for this trial is 850 mg administered thrice daily. Common adverse effects include diarrhea, nausea and abdominal pain. These can be largely mitigated by using a titration scheme and adhering to intake during or after a meal. In case the participant does not tolerate 3 tablets per day, the dose may be decreased to the maximum tolerable dose. Compliance and adherence to the study medication will be monitored through a diary. Following the conclusion of the clinical trial, the attending neurologist could consider the off-label prescription of metformin depending on the outcomes of the study.

### 2.5 Outcome measures

#### 2.5.1 Primary outcome measure

The primary outcome is the change in T25FW from baseline to 96 weeks of treatment.

#### 2.5.2 Secondary outcome measures

Clinically relevant outcome measures will be assessed as secondary outcomes: Expanded Disability Status Score (EDSS) for general disability, Symbol Digit Modalities Test (SDMT) as a measure for information processing speed and 9-hole Peg test (9HPT) as a measure of hand function.

Furthermore, imaging outcome measures include brain MRI volumetrics (change in whole brain volume and gray matter volume, change in T2 lesion volume, change in T1 lesion volume) along with MRI DTI. Both volumetric MRI and DTI metrics are used as surrogate outcomes for disability in clinical trials (64, 65) and correlate well with disability progression in clinical practice (66, 67). For the DTI analysis, the following parametric maps will be extracted: fractional anisotropy (FA), mean diffusivity (MD) and radial diffusivity (RD). Measurements will encompass both white matter and lesion areas, with a voxel-wise longitudinal evaluation being conducted.

#### 2.5.3 Tertiary outcome measures

Cost-effectiveness analysis of the metformin treatment will be conducted based on survey data on health-related quality of life, caregiver strain and healthcare resource usages. We adopt the societal perspective and make use of existing tools, such as a burden-of-illness questionnaire (69), the EQ-5D-5L, the Multiple Sclerosis Impact Scale (MSIS-29) and the Caregiver Strain Index (CSI).

As patients may not progress in all domains, a recently described composite outcome, the Overall Disability Response Score (ODRS) (70) will be evaluated as an additional exploratory outcome. Another clinical exploratory outcome will be the 2-minute walking test, as not all patients may be able to undertake this endurance assessment over time.

Susceptibility-weighted imaging will be analyzed in both the treatment and placebo arm. This allows the follow up of the presence, volume, number and destructiveness of “iron rim lesions” (IRLs), also known as “paramagnetic rim lesions”. Inflammatory lesions are seen as hyperintense lesions on T2 weighted images, while chronic lesions, also called ‘smoldering, paramagnetic or iron-rim lesions’ can be seen on T2* images as a hypo-intense rim, representing iron-loaded microglia or macrophages (25, 71–73). Together with DTI, this MRI technique offers valuable insights into the impact of metformin on MS disease mechanisms that may not be fully captured by solely assessing brain volume.

#### 2.5.4 Substudies

Study patients will be asked to participate in optional substudies. Separate informed consent forms will be used to obtain written informed consent of the patient.

##### 2.5.4.1 Adherence monitoring

This substudy is designed to monitor adherence to the study drugs through an online app (Remecare®). In case patients are unable to use an electronic device or are unwilling to use the online app, a paper diary will be provided to track study medication intake and adherence.

##### 2.5.4.2 Centralized biorepository for biomarkers

In addition to clinical, conventional and non-conventional imaging outcome measures, biomarkers and biobanking of samples for later analysis are crucial components of a clinical trial in PMS, to improve the understanding of underlying disease and therapeutic mechanisms. In this substudy we will analyze neurofilament light chain and glial fibrillary acidic protein on the SIMOA platform within the study population. Besides, a biorepository will be set up for future- and re-use of the collected biological samples, including serum, plasma and DNA. This supplementary blood sample collection is scheduled to coincide with the routine blood sampling intervals conducted for safety monitoring and routine care.

## 3 Analysis

A summary of baseline data with comparison of demographics (age, sex, ethnicity), clinical tests at baseline, concomitant therapies and disease duration between the metformin and placebo arm will be presented. For the health economic analysis, marital status, household composition/living situation and educational level will be recorded. In addition, a CONSORT flow diagram (74) will be produced to summarize the number of patients at each stage (eligibility, randomization, allocation, discontinuation and follow-up). Adverse events (AE), Serious adverse events (SAE) and Suspected Unexpected Serious Adverse Reaction (SUSAR) will be summarized per arm using descriptive statistics.

Analysis will be conducted based on the intention-to-treat population. If the data exhibit a normal distribution, the primary analysis will entail in first instance (to align with sample size calculation) an independent samples t-test to compare the alteration in T25FW from baseline to the end of the 96-week treatment period between the metformin and placebo arm. A secondary sensitivity analysis will include a linear regression model with 96-week T25FW as outcome and baseline T25FW as predictor, plus a linear mixed model with T25FW at all-time points as outcome (both models can take other confounders into account) to get a more precise estimate of the treatment effect. Similar linear regression and linear mixed models will be used to analyze the secondary and clinical tertiary outcomes at 96 weeks and over time. The cost effectiveness analysis will use the societal perspectives, including both direct and indirect, medical and non-medical costs, collected through questionnaires. Main health outcome will be Quality Adjusted Life-Years (QALYs), but other health outcomes including MSIS-29 will also be considered. The return of investment will be assessed using the incremental cost-effectiveness ratio and incremental net monetary benefit.

A per protocol-analysis will be done to evaluate the sensitivity of the main results. Use of at least ≥80% of study drug will be regarded as being compliant to study drug. Study drug adherence will be reported as mean with SD or median with interquartile range.

## 4 Data management and monitoring

An electronic case report form (eCRF) in Redcap will be used by all participating sites to collect the individual patient data in accordance with the trial protocol. This data will be stored on a dedicated storage server located at the Sponsor’s premises and retained for 25 years. Clinical trial monitoring will be performed by the study sponsor according to a prespecified monitoring plan.

### 4.1 Patient protection

Confidentiality will be maintained, and the trial is compliant with the requirements of the Belgian and European Privacy legislation (https://www.dataprotectionauthority.be/). The clinical trial will comply with the tenets of the Declaration of Helsinki and will be performed in accordance with ICH-GCP guidelines and all applicable laws and regulations. An independent data monitoring committee (DMC) will assess the progress of the clinical trial and safety data in accordance with a written DMC charter.

### 4.2 Informed consent

Qualified study personnel will provide patients with comprehensive information regarding the study, including its purpose, procedures, potential risks, benefits, and alternative options for participation. Prospective study participants will have the opportunity to ask questions and will be given sufficient time to consider participation. A printed information and consent form will be provided. Only after signing the information and consent form, the patient can be included in the study. The right of a participant to refuse or stop (withdraw of consent) participation without providing reasons will always be respected in every participating center. Upon a patient’s agreement to participate in the clinical trial, the patient’s caregiver will be notified of the possibility to take part in the clinical trial to evaluate caregiver strain. If a caregiver decides not to participate, the subject can still be enrolled in the clinical trial.

### 4.3 Dissemination

The results of the clinical trial will be disseminated as widely as possible, to the scientific community, the patient community and the general public. This includes press releases, social and classical media, presentations at scientific conferences and meetings, publications in international peer-reviewed journals.

## 5 Discussion

Presented here is the study protocol of a phase II clinical trial aimed at evaluating the effectiveness and safety of metformin as add-on treatment in patients with non-active PMS. This patient group holds a substantial unmet need for treatments that promote restoration and neuroprotection. Recently, metformin was shortlisted by the UK MS society expert consortium as a highly promising repurposed drug and recommended for immediate use in clinical trials focusing on PMS, due to its rejuvenating properties (75). Previous studies have provided preliminary data on mechanisms of action, safety and feasibility of the use of metformin in MS. These studies encompass preclinical animal models, RRMS patients and RRMS patients who presented either metabolic syndrome and/or type 2 diabetes mellitus (49, 50, 52).

Currently, several trials aiming to further investigate the safety and efficacy of metformin in multiple sclerosis are in the recruitment phase (**Table 1**). Our study aims to provide evidence for the efficacy of metformin specifically in PMS patients, by focusing on both clinical and cost-related outcome measures. By also collecting MRI data and blood samples, we aim to improve our understanding of the potential modes of action of metformin in MS. Contrary to other ongoing trials, we aim to explore remyelination, neuroprotection and smoldering inflammation in PMS patients using advanced imaging techniques, including DTI and SWI.

This study holds a notable advantage in its utilization of metformin as add-on treatment, allowing patients who are already on stable disease-modifying therapies to continue their treatment. This reflects the common clinical practice where patients with PMS can continue using a DMT, despite clinical progression without visible inflammatory disease activity on MRI. The use of T25FW as the primary outcome measure is an additional strength, as it is more sensitive to detect changes over time than the EDSS. Another advantage is the investigation of the cost-effectiveness of the intervention. While great effort is being made in investigating new molecules to slow down disease progression, development of pharmaceutics remains a time-consuming and costly undertaking. By repurposing metformin, an off-patent drug with evidence of safety in humans, blood-brain barrier penetrance and demonstrated efficacy in preclinical studies, clinical trials can be conducted quickly and affordably. Additionally, considering the low cost of metformin, even a small effect-size will prove the cost-effectiveness of this treatment.

In conclusion, MACSiMiSE-BRAIN has the potential to demonstrate the clinical benefit of metformin as an add-on treatment in PMS patients. Considering the lack of treatment options to halt disability progression, the aim of this study is to investigate a potential safe and effective treatment option for PMS, while also unravelling the underlying disease-mechanisms of disability progression.

## Ethics statement

All subjects have to provide written informed consent prior to inclusion in the study. The clinical trial was approved by regulatory authorities and ethical committee after submission to the European Clinical Trials Informations System (EUCT number: 2023-503190-38-00, Date of approval: April24, 2023).

## Author contributions

A-VD: Conceptualization, Formal analysis, Funding acquisition, Investigation, Methodology, Project administration, Visualization, Writing – original draft, Writing – review & editing, Validation. EV: Conceptualization, Formal analysis, Investigation, Methodology, Project administration, Visualization, Writing – original draft, Writing – review & editing. VP: Conceptualization, Investigation, Supervision, Writing – original draft, Writing – review & editing. LW: Formal analysis, Investigation, Supervision, Writing – original draft, Writing – review & editing. MC: Conceptualization, Investigation, Supervision, Writing – original draft, Writing – review & editing. GL: Conceptualization, Investigation, Supervision, Writing – original draft, Writing – review & editing. MD’H: Conceptualization, Investigation, Supervision, Writing – original draft, Writing – review & editing. MB: Formal analysis, Supervision, Writing – original draft, Writing – review & editing. ER: Conceptualization, Formal analysis, Methodology, Supervision, Writing – original draft, Writing – review & editing. ML: Formal analysis, Supervision, Writing – original draft, Writing – review & editing. MBD’H: Investigation, Supervision, Writing – original draft, Writing – review & editing. JD: Conceptualization, Investigation, Methodology, Supervision, Writing – original draft, Writing – review & editing. TR: Conceptualization, Investigation, Methodology, Project administration, Supervision, Writing – original draft, Writing – review & editing. BW: Conceptualization, Formal analysis, Funding acquisition, Investigation, Methodology, Project administration, Resources, Supervision, Validation, Visualization, Writing – original draft, Writing – review & editing.

## MACSiMiSE-BRAIN study group collaborators

Elke Smits, Antwerp University Hospital, Clinical Trial Center, Antwerp, Belgium; Iris Verhagen, Antwerp University Hospital, Clinical Trial Center, Antwerp, Belgium; Valerie Bastiaens, Antwerp University Hospital, Pharmacy, Belgium; Kim Claes, Clinical Trial Center, Antwerp, Belgium; Lauren Meers, Antwerp University Hospital, Clinical Trial Center, Antwerp, Belgium; Christella Ukunda, Noorderhart Maria Hospital, Department of Neurology, Pelt, Belgium; Lynn Delameilleure, AZ Sint Jan, Department of Neurology, Bruges, Belgium; Liesbeth Laloo, AZ Sint Jan, Department of Neurology, Bruges, Belgium; Ann Van Remoortel, National Multiple Sclerosis Center, Melsbroek, Belgium; Laetitia della Faille, National Multiple Sclerosis Center, Melsbroek, Belgium; Helen Bachmann, University Hospital Ghent, Belgium.
