# Supplementary material for: A metformin add-on clinical study in multiple sclerosis to evaluate brain remyelination and neurodegeneration (MACSiMiSE-BRAIN): study protocol for a multi-center randomized placebo controlled clinical trial
Source: Front Immunol. 2024 Feb 21;15:1362629. doi: 10.3389/fimmu.2024.1362629 (PMC11046490; doi:10.3389/fimmu.2024.1362629)
Supplement: Supplementary file 1 [file DataSheet_1.pdf]

frontiers

# *Supplementary Material*

# A Metformin Add-on Clinical Study in Multiple Sclerosis to Evaluate Brain Remyelination And Neurodegeneration (MACSiMiSE-BRAIN): study protocol for a multi-center randomized placebo controlled clinical trial

**De Keersmaecker A, Van Doninck E., Popescu V., Willem L., Cambron M., Laureys G., D' Haeseleer M., Bjerke M., Roelant E., Lemmerling M., D'hooghe M., Derdelinckx J., Reynders T., Willekens B.*, on behalf of the MACSiMiSE-BRAIN study group**

*** Correspondence:** Prof. Dr. Barbara Willekens: barbara.willekens@uza.be

## 1 Supplementary Table

**Table 3** Overview of the study schedule

| **Procedures** | **Screening** | **Baseline** | **Study visits/ telephone visits*** | | | | | | | **Final visit** |
|---|---|---|---|---|---|---|---|---|---|---|
| Visit number | V1 | V2 | V3* | V4 | V5* | V6 | V7* | V8 | V9* | V10 |
| Time | -4W | 0W | W12 | W24 | W36 | W48 | W60 | W72 | W84 | W96 |
| Informed consent | X | | | | | | | | | |
| Medical history | X | | | | | | | | | |
| Concomitant medication/ treatment | X | X | X | X | X | X | X | X | X | X |
| Physical examination | X | X | | X | | X | | X | | X |
| Eligibility assessment | | X | | | | | | | | |
| Randomization | | X | | | | | | | | |
| IMP | | X | | X | | X | | X | | |

| | | | | | | | | | | |
|---|---|---|---|---|---|---|---|---|---|---|
| Compliance | | | X | X | X | X | X | X | X | X |
| Adverse events | | | X | X | X | X | X | X | X | X |
| Neurological examination and EDSS | X | | | X | | X | | X | | X |
| T25FWT | X | | | X | | X | | X | | X |
| SDMT | X | | | X | | X | | X | | X |
| 9HPT | X | | | X | | X | | X | | X |
| 2MWT | X | | | X | | X | | X | | X |
| Brain MRI | X | | | | | X | | | | X |
| Laboratory | X | | | X | | X | | X | | X |
| Samples for biorepository[#] | X | | | X | | X | | X | | X |
| EQ5D-5L | X | | | X | | X | | X | | X |
| MSIS-29 | X | | | X | | X | | X | | X |
| Health economics questionnaire | X | | | X | | X | | X | | X |
| CSI | X | | | X | | X | | X | | X |

[#]Optional substudy

IMP: Investigational Medicinal Product
